# Investigating the early impact of the Trump Administration’s Global Gag Rule on sexual and reproductive health service delivery in Uganda

**DOI:** 10.1371/journal.pone.0231960

**Published:** 2020-04-28

**Authors:** Margaret Giorgio, Fredrick Makumbi, Simon Peter Sebina Kibira, Suzanne Bell, Selena Anjur-Dietrich, Elizabeth Sully

**Affiliations:** 1 Guttmacher Institute, New York, New York, United States of America; 2 School of Public Health, College of Health Sciences, Makerere University, Kampala, Uganda; 3 Johns Hopkins Bloomberg School of Public Health, Baltimore, Maryland, United States of America; Syracuse University, UNITED STATES

## Abstract

**Background:**

The Global Gag Rule (GGR), reinstated by President Trump in January 2017, makes non-U.S. non-governmental organizations ineligible for U.S. foreign assistance if they provide access to or information about abortion. While evidence suggests previous iterations of the GGR negatively impacted sexual and reproductive health outcomes, no studies have quantitatively assessed the impacts of the Trump administration’s GGR.

**Methods:**

We constructed a panel dataset of facilities (76% public) using 2017/2018 Performance Monitoring and Accountability 2020 service delivery point (SDP) surveys in Uganda. Based on information from stakeholder meetings, we classified districts as more or less exposed to the GGR; 45% (N = 34) of study districts were classified as “more exposed”, which corresponded to 145 “more exposed” and 142 “less exposed” health facilities in our sample. We assessed changes in provision of long-acting reversible contraceptives, contraceptive stock-outs, mobile outreach services, engagement with community health workers (CHWs), service integration, and quality of care from 2017 (pre-GGR) to 2018 (post-GGR). Multivariable regression models were estimated, and difference-in-differences impact estimators were determined by calculating predicted probabilities from interaction terms for exposure and survey round.

**Findings:**

We observed no immediate impact of the GGR on the provision of long-acting reversible contraceptives, contraceptive stock-outs, mobile outreach services, service integration, or quality of care. We did observe a significant impact of the policy on the average number of CHWs, with “more exposed” facilities engaging 3.8 fewer CHWs post-GGR (95% CI:-7.31,-0.32).

**Conclusions:**

The reduction in CHWs could reduce contraceptive use and increase unintended pregnancies in Uganda. The lack of other significant findings may not be surprising given the short post-GGR observation window. Rapid organizational responses and stopgap funding from foreign governments may have mitigated any immediate impacts on service delivery in the short term. The true impact may not be felt for many years, as stopgap funding potentially ebbs and service providers adapt to new funding environments.

## Introduction

In January 2017, the United States (U.S.) instated an expanded version of the Mexico City Policy, commonly referred to as the Global Gag Rule (GGR). This policy makes non-U.S. non-governmental organizations (NGOs) ineligible for U.S. foreign assistance if they provide abortion services, or provide abortion-related counseling, referrals, advocacy, or information. Previous iterations of the policy have been in place under every Republican administration since President Reagan. While the most recent version of the policy under President George W. Bush applied restrictions to family planning (FP) and reproductive health services only (~$600 million annually, fiscal years 2002–2009), the Trump administration expanded the GGR’s reach to apply restrictions to funding for virtually all U.S. health programs (~$8.8 billion, fiscal year 2017).[1]

The policy could affect sexual and reproductive health (SRH) services through two pathways. First, non-U.S. NGOs that do not sign the policy and lose U.S. funding may experience reduced service provision, staff loss, program terminations, or decreased capacity to support public sector services.[2–4] Second, the policy may produce a “chilling effect”, leaving providers and advocates who sign unwilling to provide other SRH services for fear of breaching the policy.[2,3] While the policy is targeted at non-U.S. NGOs, it has the potential to impact SRH services provided by both the public and private sector. This is due to the fact that, in many countries, public sector providers rely on non-U.S. NGOs for technical support, additional staffing, and training. Further, public facilities may not have the capacity to absorb the needs of patients who can no longer access family planning services that were previously provided by non-U.S. NGOs.[3]

**Fig 1:**
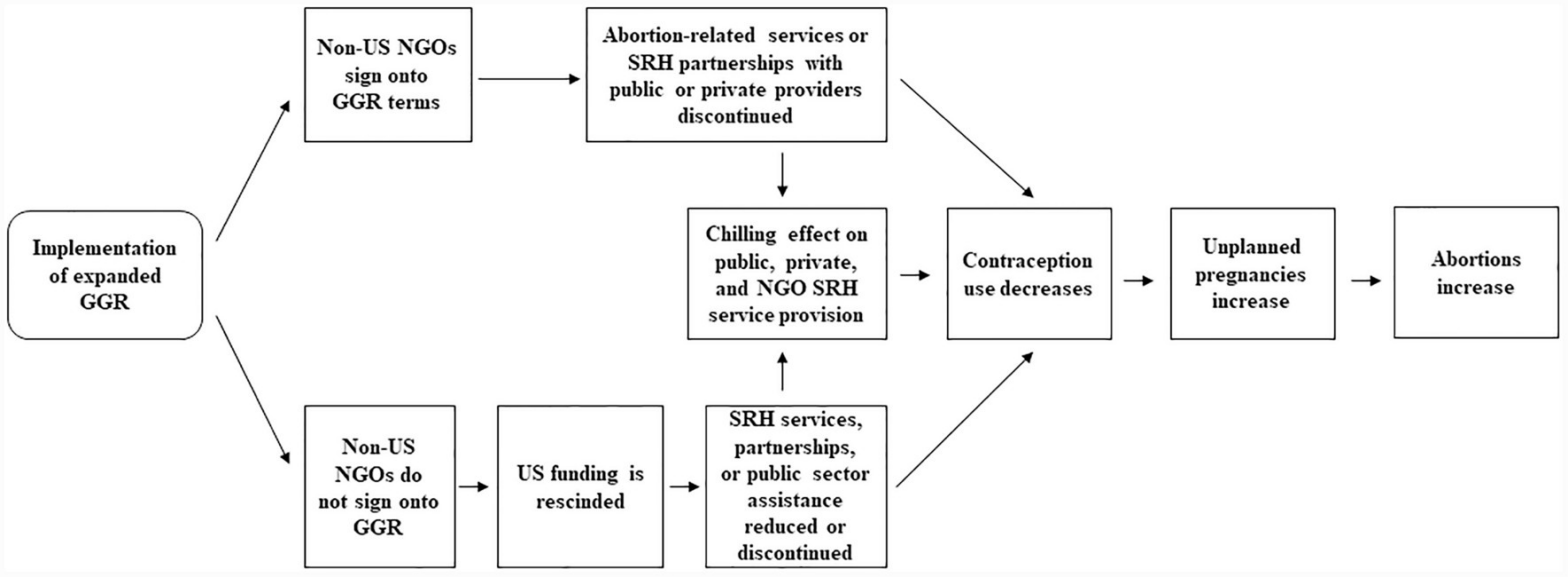
Hypothetical causal pathway for the impact of the Global Gag Rule on service delivery and women’s SRH outcomes.

Change in service delivery is the first step in a hypothetical causal pathway that could lead to negative SRH outcomes among women: if access to modern contraceptive methods decreases, women’s use of modern contraceptives will decrease and unintended pregnancies will increase, which ultimately creates the potential for induced abortions to increase (Fig 1).

Little quantitative evidence exists on the policy’s impact in sub-Saharan Africa. A study by Jones et al. 2015 evaluated the impact of the Bush administration’s iteration of the GGR in Ghana and found an overall decrease in contraceptive provision, as well as reduced contraceptive use and increased rates of unintended pregnancy among rural women.[5] Other quantitative evidence suggests that country-level abortion rates increased when the Bush administration’s GGR was in effect in countries that received large amounts of U.S. foreign aid [6,7], and these rates subsequently decreased during the Obama administration [8].

To our knowledge, no studies have quantitatively assessed the impact of the Trump administration’s GGR. However, several qualitative reports suggest providers have recently reduced or altered SRH services due to GGR-related funding gaps, confusion about the policy’s scope, and burdens associated with implementation of the policy.[3,9,10] In the specific context of Uganda, some early qualitative evidence suggests that the policy has introduced complications into partnerships between signing and non-signing organizations.[3] This evidence also suggests the presence of a “chilling effect” in Uganda, as some GGR-compliant organizations are avoiding activities that they feel unable to adequately distinguish from abortion-related care (e.g. discontinuing training on using misoprostol for post-partum hemorrhage).[3]

The policy’s impacts on SRH service delivery may be particularly acute in Uganda. Unmet need for modern contraception and the proportion of unintended births are relatively high in Uganda compared to all low- and middle-income regions (unmet need: 20% vs.13%; unintended births: 41% vs. 23%).[11,12] Further, an estimated 14% of all pregnancies in Uganda end in abortion.[13] Safe abortion care is highly restricted in Uganda, and conflicting policies around abortion create a complicated legal environment for providers.[14]

The loss of USAID funding has the potential to place Uganda’s FP service provision in a precarious and vulnerable state. The Uganda Ministry of Health (MOH) estimated total funding need for the family planning program and contraceptive commodities in fiscal year 2017 at $19.8 million and $25.5 million, respectively,[15,16] and the government pledged under 10% of these costs in 2018. While data on total family planning expenditures in Uganda is not publically available,[16] USAID is by far the largest international donor to Uganda’s FP budget, contributing 90% of all international disbursements in fiscal year 2016.[16,17] Further, non-U.S. NGOs, who are major recipients of this US funding, play an important role by delivering over half of Uganda’s FP services.[3] While the Ugandan public health system has increased its capacity to provide SRH services in recent years, non-U.S. NGOs continue to provide crucial support to public sector facilities in the form of commodities, training, mobile outreach visits, and other technical assistance.[3,18–20]

The aim of this paper is to investigate whether the expanded GGR has impacted SRH service delivery in Uganda in the first year after the policy’s implementation. We hypothesize that the loss of U.S. funding and disrupted partnerships that resulted from the GGR’s reinstatement have led to declines in mobile outreach visits, community health worker (CHW) engagement, FP service integration, and contraceptive availability. We hypothesize that long acting reversible contraceptives (LARCs) may be particularly vulnerable to the policy’s impact; much of the U.S. funding to foreign NGOs in Uganda was in support of direct LARC provision as well as programs allowing these NGOs to provide technical assistance, training, and other support to public sector facilities for the purpose of LARC provision. Using data from the Performance Monitoring and Accountability 2020 (PMA2020) survey in Uganda, we investigate changes in several SRH service delivery outcomes from early 2017 (pre-GGR implementation) to 2018 (after the policy officially took effect in July 2017) based on policy exposure, thereby providing a quantitative assessment of the early impact of the expanded GGR on SRH service delivery in Uganda.

## Methods

### Data source and sampling design

This study utilizes data from the 2017 and 2018 PMA2020 service delivery point (SDP) and female surveys, which were conducted face-to-face in April-May 2017 and May-June 2018 using Open Data Kit (ODK) software.[21] PMA2020 uses a two-stage cluster sampling design, with urban-rural and ten statistical regions as the strata, resulting in a nationally representative collection of 110 enumeration areas (EAs). For the SDP sample, all public facilities serving selected EAs were surveyed, regardless of whether the SDP was located within the geographical boundaries of the EA. For private facilities, interviewers mapped all facilities within the EA and surveyed up to three; in the event there were more than three private facilities, study staff randomly selected three. For the female questionnaire, interviewers mapped and listed all households within an EA, and 44 households were randomly selected to participate in the survey. All women aged 15–49 who slept in the household the previous night or were usual members of the household were invited to participate in the survey.

**Fig 2:**
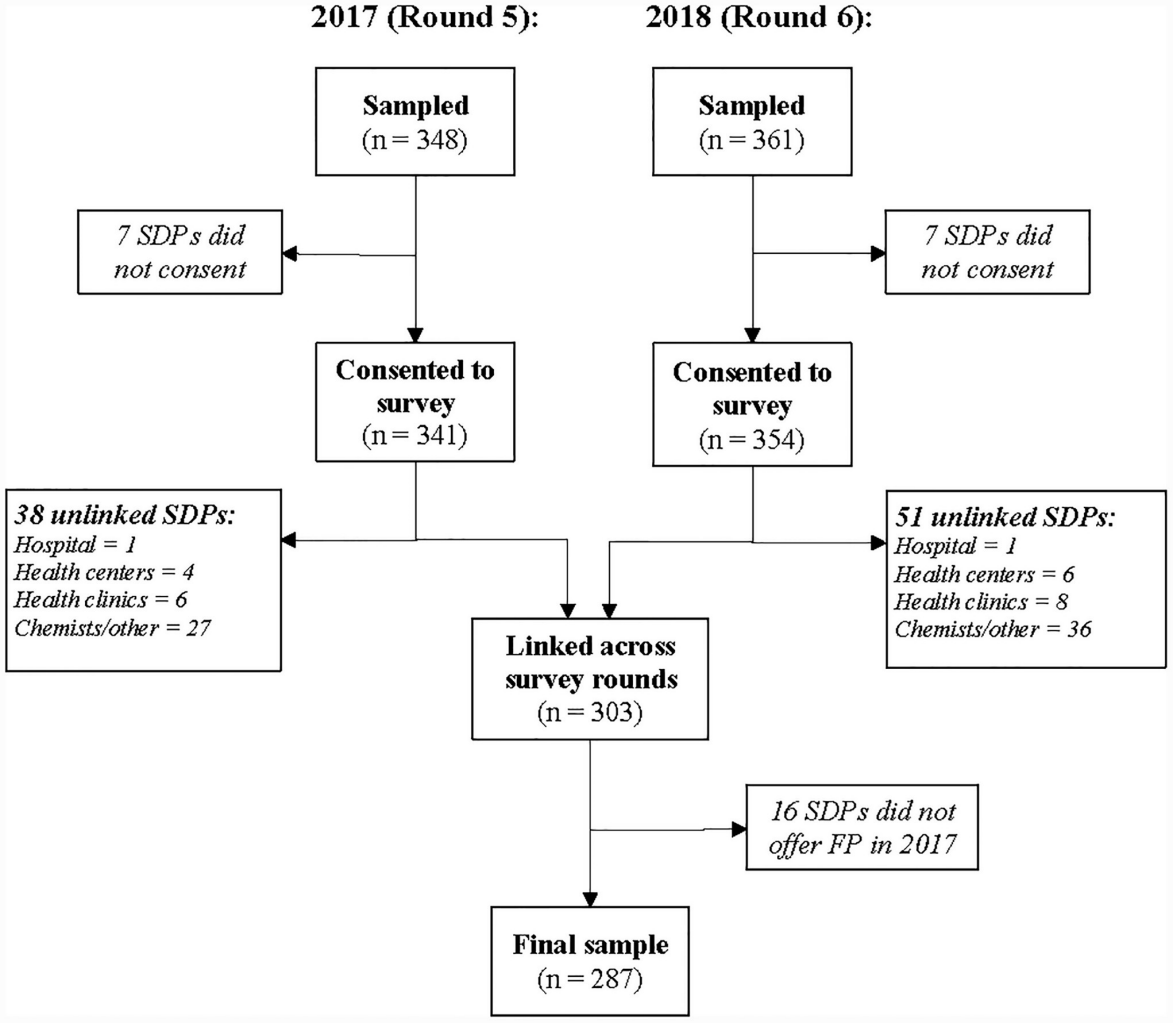
Selection process for analytic sample.

To generate a variable that measures exposure to the GGR, in April 2018 we met with service providers, NGOs, government agencies, and advocates to collect information on the implementation of the policy. We collected information on changes in service delivery and funding due to the enactment of the GGR, funding allocations by year from USAID and other donors, and information on other contextual changes impacting the service delivery environment unrelated to the GGR. We constructed a panel dataset by matching SDPs across the 2017 and 2018 survey rounds (Fig 2). The PMA2020 sample included 348 SDPs in 2017 and 361 SDPs in 2018. After removing facilities that did not provide consent or were not surveyed in both years, we matched 303 SDPs. We further excluded sixteen SDPs because they did not offer FP in 2017, which resulted in a final analytic sample of 287 facilities (83% of SDPs sampled in 2017, 80% of SDPs sampled in 2018).

The Institutional Review Boards of [names redacted for blinding] provided final approval.

### Measures

Exposure to the GGR was classified at the district level. Study stakeholder meetings revealed that signing organizations in Uganda had not experienced major changes in service delivery or partnerships, largely because these organizations were not engaged in safe abortion care or advocacy prior to the policy’s reinstatement. Therefore, our exposure variable only captures changes among non-signing organizations, who consequently were ineligible for further U.S. funding.

In Uganda, two large non-U.S. NGOs did not sign the policy and lost U.S. funding. Since we did not have access to specific funding levels that were lost, we instead interviewed organization staff to understand how the loss of U.S. funding had impacted services and activities. One organization lost a large proportion of funding for their mobile outreach program, which provided additional trained staff, information, services, contraceptives, and supplies to public facilities and other venues in communities where these services were not adequately available. As a result of the GGR, the organization was forced to reduce the capacity or close several of these outreach teams. Another organization reported that several USAID-funded programs ended prematurely after they refused to sign the policy. These programs provided training and technical assistance for public facilities, community education on family planning methods, and other SRH related advocacy. These program changes were associated with lost funding for staff salaries. Lastly, one facility closure was also reported.

We collected detailed geographic information on where these programs were operating, where there were reductions in mobile outreach coverage, and where other SRH services changes occurred. We then coded districts as being “more exposed” to the expanded GGR if at least one of these program changes occurred within the district; “less exposed” districts experienced no weakening of mobile outreach, facility, or program coverage. This process resulted in 45% (N = 34) of districts being classified as “more exposed” to the GGR, and the remaining 55% (N = 41) classified as “less exposed”. SDPs were linked to their respective districts, which resulted in 145 “more exposed” SDPs and 142 “less exposed” SDPs in the final sample. The SDPs in both the “more” and “less” exposed groups represent a national sample of all health facilities, not just those that refused to sign the policy and lost funding. However, given the ways in which the non-signing NGOs support public sector facilities and services, we expect that any potential policy impacts would observable across all facility types in our sample.

We use the terms “more” and “less” exposed to account for the difficulty in capturing all possible mechanisms for impacts on service delivery in Uganda. These include other types of impacts on organizations that may not have been identified in our key informant interviews, as well as countrywide “chilling effects”, whereby misunderstandings of the policy’s applicability or scope by family planning providers may cause them to be less likely to engage in outreach activities, pursue partnerships with other organizations, or provide certain kinds of SRH services, such as post-abortion care.

We investigate changes in several different SRH service delivery outcomes. We included dichotomous indicators for whether each SDP provides two types of LARCs (IUDs and implants, separately). Any recent stock-outs of a modern contraceptive method (contraceptive pills, emergency contraception, male or female condoms, IUDs, implants, and subcutaneous/intramuscular injectables) was measured by coding SDPs as “1” if they reported being stocked out of at least one method in the last three months and “0” if they had experienced no recent stock-outs. Community and mobile outreach indicators measured whether the SDP provided any FP services through CHWs and whether the SDP received support from FP mobile outreach teams. For each outcome, we first constructed a dichotomous variable that indicates whether the SDP had provided FP services through any CHWs/received any mobile outreach visits in the last 12 months. Second, we examined these indicators as continuous variables measuring the number of CHWs engaged/mobile outreach visits at each SDP in the prior 12 months. Two dichotomous service integration variables were included that indicated whether each SDP offered 1) integrated FP and post-abortion care (PAC) services, and 2) integrated FP and HIV services.

Finally, we created a quality of care index for each SDP by adapting a methodology developed by researchers at Johns Hopkins University, a detailed explanation of which is provided elsewhere.[22] In brief, using 122 dichotomous indicators from the SDP survey, six indices are created based on the Bruce-Jain framework for FP service quality, representing method choice, information given to users, technical competence, interpersonal relations, continuity mechanisms, and constellation of services.[22,23] The total quality index score is the sum of the six indices (maximum value = 122), which is then normalized by computing z-scores. For this sample, the mean 2017–2018 pooled quality index score was 1.00 (SD = 3.49, range -7.44–6.92).

Potential confounding variables included facility type (hospitals, health center levels IV-II, health clinics, pharmacies/chemists) and managing authority (government, NGO, faith-based organization, private). Managing authority was further recoded as public (government) and non-public (all else). In addition, using data from the female questionnaire, we calculated the modern contraceptive prevalence rate (mCPR) for each district in 2017 to account for demand-driven SRH service provision that is unrelated to the implementation of the GGR. An indicator for the proportion of SDPs in 2017 within each district that offered IUDs was also included to account for local differences in LARC availability.

### Statistical analysis

In order to determine whether the “less-exposed” group of SDPs represents a reasonable counterfactual in this quasi-experiment study, we used bivariate chi-squared tests and t-tests to examine differences in facility characteristics, community-level controls, and the study’s key outcomes by exposure status during the baseline period (2017). The impact of the GGR was isolated using a difference-in-differences (DID) approach. Multivariable regression models for each outcome were estimated using the following formula:

$$Y_{ij}=E_{i}+T_{j}+\left(E_{i}*T_{j}\right)+F_{i}+C_{ij}+{Ɛ}_{ij}$$

where *Y*_*ij*_ represents the SRH service provision outcome measure for SDP *i* in survey round *j*, *E*_*i*_ represents exposure to the GGR (0/1) for SDP *i*, *T*_*j*_ represents the survey round (2017/2018), *E*_*i*_ *T_*j*_ represents the interaction of SDP *i’s* exposure to the GGR in survey round *j*, *F*_*i*_ represents the facility type for SDP *i*, and *C*_*ij*_ represents the community-level controls for SDP *i* in survey round *j*. The DID estimator of impact was determined by calculating predicted probabilities for each exposure group and survey round using the interaction term. Dichotomous outcomes were fitted using logit models, the continuous quality of care score outcome model was fit using OLS regression, and the mobile outreach and CHWs count data outcomes were fitted using zero-inflated Poisson regression models with robust, clustered standard errors for each SDP. For all analyses, *P* values were based on two-tailed significance tests, with alphas less than 0.05 considered statistically significant. Analyses were performed using Stata version 15.0 (StataCorp LP, College Station, TX).

## Results

Descriptive characteristics for facilities and baseline values of outcome variables are shown in Table 1. Due to sample design, the majority of sampled SDPs were public facilities (76%). Facility type distributions were as follows: 15% hospitals, 68% health center types IV-II, 6% health clinics, and 11% pharmacies/chemists. In 2017, average district-level mCPR was 29% (range 0–60%), and the average EA-level proportion of facilities that offered IUDs was 47% (range 0–100%).

**Table 1:** Baseline differences between service delivery points more and less exposed to the GGR, 2017.

	Total		More Exposed		Less Exposed		p-value
	(N = 287)		(N = 145)		(N = 142)		
**Facility characteristics**							
Facility type, n(%)							0.54
Hospital	43	*(15%)*	23	*(16%)*	20	*(14%)*	
Health Center IV	54	*(19%)*	24	*(17%)*	30	*(21%)*	
Health Center III	76	*(26%)*	40	*(28%)*	36	*(25%)*	
Health Center II	65	*(23%)*	29	*(20%)*	36	*(25%)*	
Health Clinic and Other	18	*(6%)*	12	*(8%)*	6	*(4%)*	
Pharmacy or Chemist	31	*(11%)*	17	*(12%)*	14	*(10%)*	
Region, n(%)							p<0.001
Central 1	23	*(8%)*	17	*(12%)*	6	*(4%)*	
Central 2	38	*(13%)*	28	*(19%)*	10	*(7%)*	
East Central	44	*(15%)*	9	*(6%)*	35	*(25%)*	
Eastern	34	*(12%)*	11	*(8%)*	23	*(16%)*	
Kampala	20	*(7%)*	20	*(14%)*	0	*(0%)*	
Karamoja	14	*(5%)*	12	*(8%)*	2	*(1%)*	
North	36	*(13%)*	15	*(10%)*	21	*(15%)*	
South West	36	*(13%)*	23	*(16%)*	13	*(9%)*	
West Nile	14	*(5%)*	7	*(5%)*	7	*(5%)*	
Western	28	*(10%)*	3	*(2%)*	25	*(18%)*	
Managing authority, n(%)							0.59
Government	218	*(76%)*	106	*(73%)*	112	*(79%)*	
NGO	5	*(2%)*	2	*(1%)*	3	*(2%)*	
Faith-Based Organization	14	*(5%)*	8	*(6%)*	6	*(4%)*	
Private	50	*(17%)*	29	*(20%)*	21	*(15%)*	
**Community-level controls**							
Modern contraceptive prevalence rate (mCPR) by district, mean(range)	0.29	*(0*.*00–0*.*60)*	0.29	*(0*.*00–0*.*60)*	0.28	*(0*.*03–0*.*46)*	0.54
Proportion of facilities offering IUD by EA, mean(range)	0.47	*(0*.*00–1*.*00)*	0.49	*(0*.*00–1*.*00)*	0.45	*(0*.*00–1*.*00)*	0.29
**Contraceptive availability**							
Modern methods offered, n(%)							
Sterilization (male or female)	65	*(23%)*	34	*(23%)*	31	*(22%)*	0.74
IUDs	144	*(50%)*	75	*(52%)*	69	*(49%)*	0.60
Injectables	270	*(94%)*	137	*(94%)*	133	*(94%)*	0.77
Implants	172	*(60%)*	88	*(61%)*	84	*(59%)*	0.79
Pills	224	*(78%)*	113	*(78%)*	111	*(78%)*	0.96
Condoms (male or female)	265	*(92%)*	136	*(94%)*	129	*(91%)*	0.35
Quality of care index,† mean(range)	0.9	*(-6*.*46–6*.*92)*	0.7	*(-6*.*30–6*.*92)*	1.2	*(-6*.*46–6*.*77)*	0.45
Stock-out of any method offered last 3 months‡, n(%)	195	*(68%)*	101	*(70%)*	94	*(66%)*	0.53
**Community involvement**							
Provides family planning through CHWs§, n(%)	141	*(59%)*	68	*(58%)*	73	*(60%)*	0.79
Number of CHWs engaged§ ‡‡, mean(range)	6.2	*(0–100)*	7.2	*(0–100)*	5.2	*(0–36)*	0.14
Any mobile outreach visit in the past 12 months§, n(%)	195	*(79%)*	94	*(75%)*	101	*(83%)*	0.14
Number of mobile outreach visits in the past 12 months§ ‡‡‡, mean(range)	5.1	*(0–69)*	4.7	*(0–48)*	5.4	*(0–69)*	0.42
**Service integration**							
Offers FP and HIV services, n(%)	236	*(82%)*	119	*(82%)*	117	*(82%)*	0.94
Offers FP and PAC services§, n(%)	178	*(70%)*	85	*(66%)*	93	*(73%)*	0.28

^†^ Quality of care scores are standardized z-scores.

^‡^ Any stock-out of any family planning method in the past 3 months. Summary measure of stock-outs (iuds, injectables, implants, pills, male condoms, female condoms, emergency contraception)

^§^ Excludes chemists and pharmacies (N = 256, 128 exposed and 128 unexposed.)

^‡‡^ Total median = 2, IQR = 0–6

^‡‡‡^ Total median = 3, IQR = 1–6

Contraceptive methods most commonly offered at baseline were subcutaneous/intramuscular injectables (94%) and condoms (primarily male) (92%), and a majority of SDPs offered contraceptive pills (78%), implants (60%), and IUDs (50%). Sixty-eight percent of facilities had experienced a stock-out of at least one modern contraceptive method in the last three months. Just over half of applicable SDPs (59%) reported providing FP through CHWs, and an average of 6.2 CHWs were engaged by each facility (range:0–100). Seventy-nine percent of SDPs reported any mobile outreach visits in the last 12 months, with an average of 5.1 visits (range:0–69) per facility. Integration of FP services was common, with 82% of SDPs providing FP with HIV services, and 70% providing FP with PAC services. The mean quality of care index score at baseline was 0.9 (range -6.0–7.0).

In order to determine how balanced the “more” and “less” exposed groups were in this quasi-experimental study, all study variables were compared by exposure status at baseline (Table 1). There were no significant differences in the two groups, except by region, which was expected due to the geographic nature of the exposure variable.

**Fig 3:**
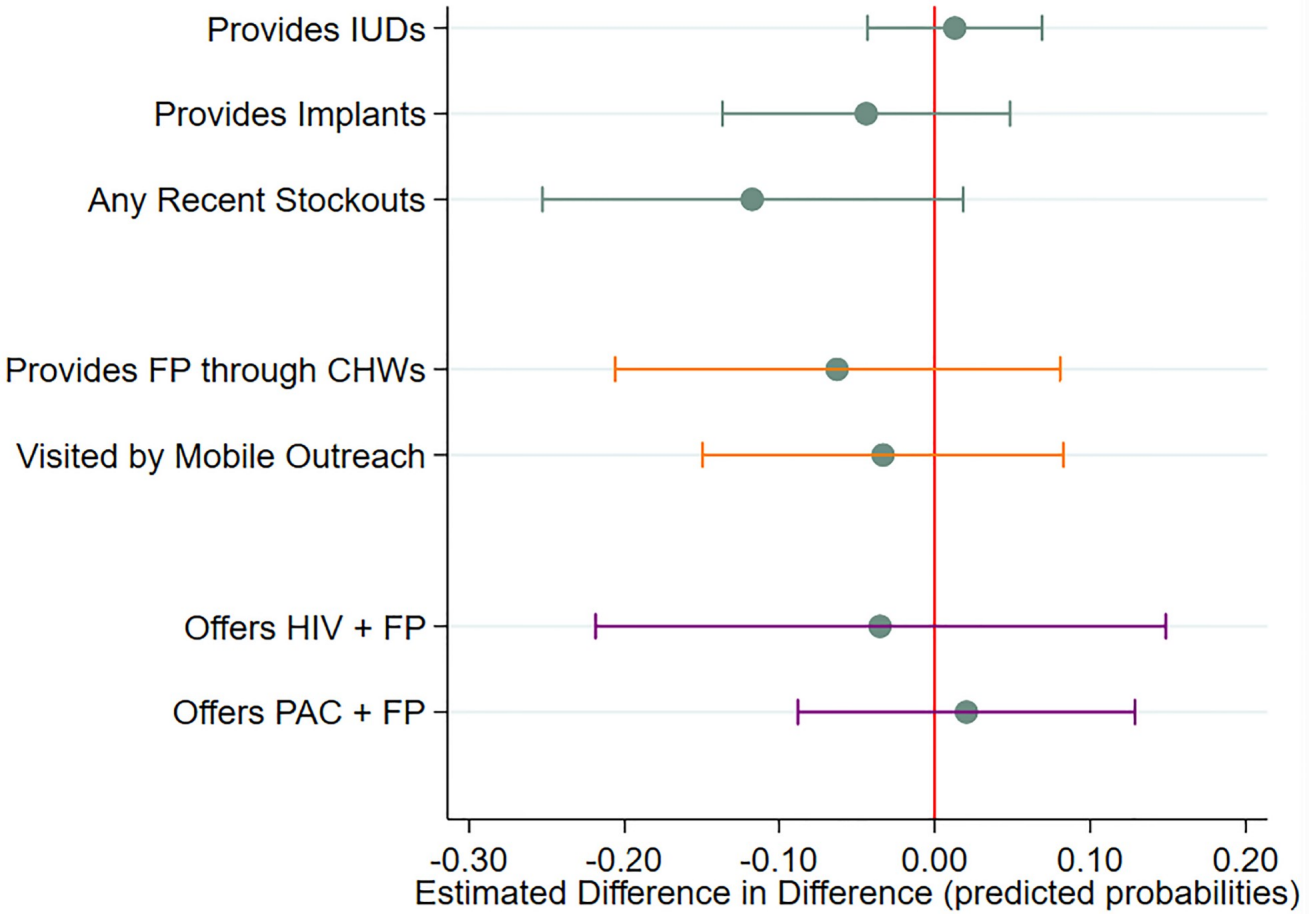
Difference in difference of selected dichotomous outcomes between more exposed and less exposed SDPs.

**Fig 4:**
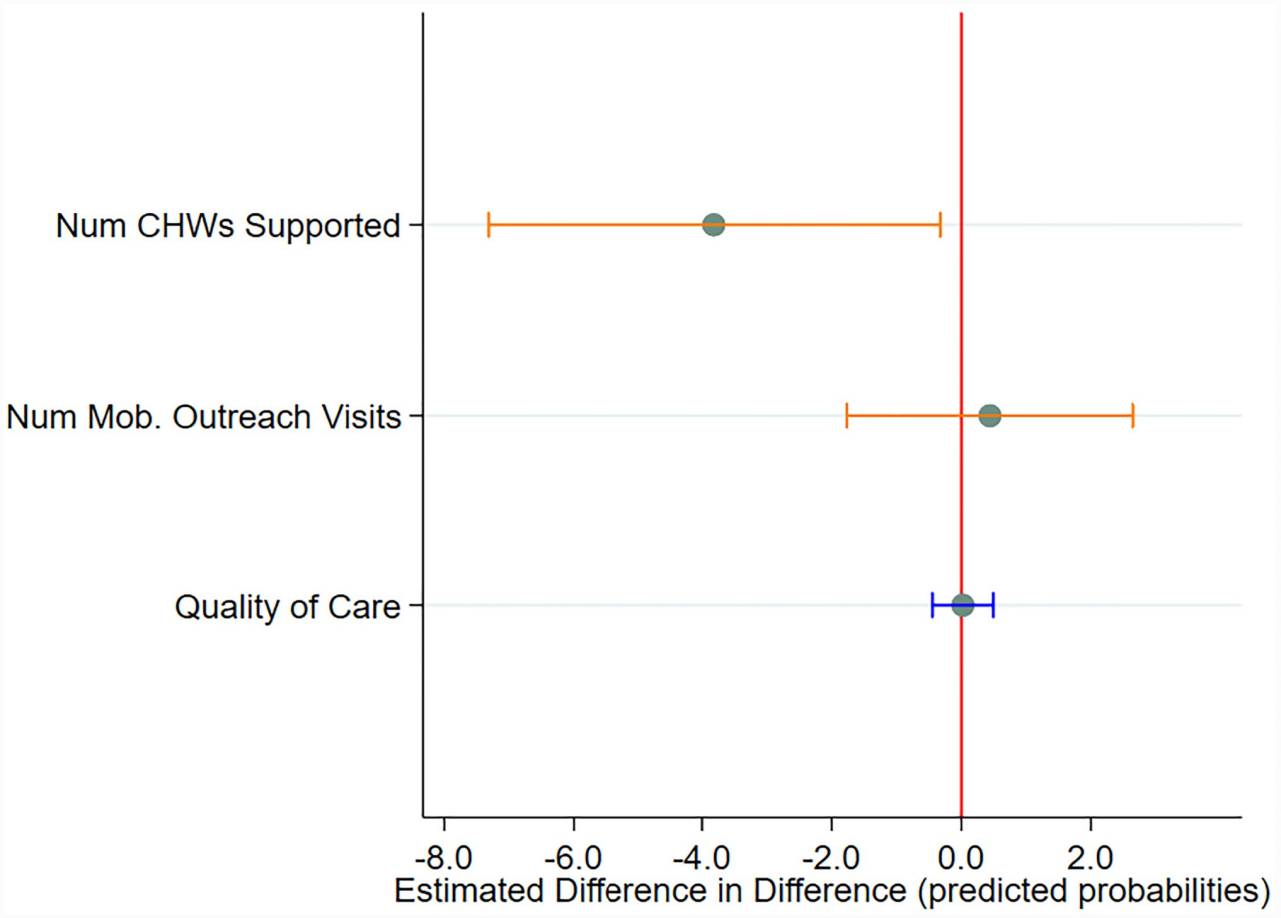
Difference in difference of selected continuous outcomes between more exposed and less exposed SDPs.

Difference-in-difference estimates for the early impact of the GGR are displayed in Figs 3 and 4. (S1 Table provides estimated adjusted proportions/means for each time-period and exposure group, difference-in-differences estimates, 95% CIs, and p-values.) After controlling for facility type, managing authority, district-level mCPR, and proportion of facilities offering IUDs, there were no statistically significant impacts for contraceptive availability, mobile outreach, service integration, or quality of care. However, a statistically significant reduction in the average number of CHWs engaged by SDPs was observed, with more exposed facilities losing 3.8 CHWs in the post-GGR period (95% CI:-7.31,-0.32) relative to the corresponding change among less exposed facilities.

## Discussion

In the first year after the implementation of the Trump-era GGR, we observed no impacts on most of our hypothesized outcomes. The only significant impact we observed was on facilities’ engagement with CHWs, with more exposed facilities deploying fewer CHWs to conduct FP services after the policy’s implementation. This result is likely due to the way that CHWs are trained and funded in Uganda. The MOH in Uganda organizes the CHWs and coordinates with lower-level health facilities to provide outreach services related to family planning use and education in the community.[24] While the MOH oversees the program, CHWs themselves are trained and deployed by disparate projects and funders.[25] This training and recruitment is largely provided by foreign NGOs, and the money to support these programs ultimately comes from foreign donors, such as USAID and DFID.[25] Therefore, these findings suggest that the funding, programmatic, and staffing decreases that non-signing organizations experienced as a result of the GGR are responsible for the differential decrease in CHWs in more exposed districts.

Reducing engagement with CHWs providing FP services has the potential to harm health outcomes in populations served. Increasing health equity and maximizing healthcare coverage are main drivers for implementing a CHW-based service delivery program,[26] and CHWs may be particularly effective in serving communities with low resources and/or high unmet need for modern contraceptives.[27–30] CHWs are an integral part of primary health care in Uganda.[31–33] CHWs are engaged by SDPs to provide FP services such as demand generation for modern contraceptives and the distribution of short-term contraceptive methods,[31,32] and previous work in Uganda has shown that FP CHWs are an effective way to increase access to modern FP methods.[27,34,35] Therefore, it is possible that this reduction in FP CHWs may ultimately reduce contraceptive use and increase unintended pregnancy rates in affected communities. Further, women in under-resourced communities could be subject to a disproportionate impact of the policy. Future work that investigates the longer-term impact of the GGR on women’s outcomes will allow us to test these hypotheses.

Stock-outs of family planning commodities may be influenced either by changes in commodity supply chains or changes in demand for commodities. Our analysis revealed that the impact on recent stock-outs of modern contraceptive methods was marginally significant with a relatively narrow confidence interval (-0.12, 95% CI:-0.25–0.02, p = 0.09), possibly suggesting that more exposed facilities may be less likely to experience stock-outs. If this reduction is not due to chance, it is unlikely that more exposed facilities benefited from increased supplies of contraceptive commodities. Instead, this result could be due to decreased demand for modern contraceptives in exposed districts, perhaps resulting from the reduction in the number of CHWs. More research is needed to better understand the relationship between the GGR and stock-outs in Uganda.

Given the rapid timeline for this study’s assessment of the impact of the GGR after its first year of implementation, it is not necessarily surprising that we did not find an early impact on the other service delivery outcomes, namely LARC provision, mobile outreach visits, FP service integration with HIV or PAC services, or FP quality of care. Previous work has demonstrated that there tends to be a lag between policy implementation and the ability to detect population-level impacts; as a result, rapid evaluation assessments have the potential to underestimate true policy impacts, as these will not have had sufficient time to develop [36–38]. To this point, policy implementation dates are routinely time lagged by one or several years in the policy evaluation literature to account for this phenomenon. [39–43] In the specific context of Uganda, we believe that rapid organizational responses may have mitigated any immediate impacts for service delivery. During the study stakeholder meetings, non-signing organizations noted that they had responded to the GGR’s reinstatement by implementing strategies to help absorb impacts that would be felt by the women accessing FP services, such as reorganizing mobile outreach teams to cover more districts. This type of organizational response may have allowed for sustained pre-GGR service delivery levels in the short-term. However, it places an enormous burden on already stretched organizations and staff, and maintaining this level of activity may prove difficult in subsequent years. In addition, as an immediate response to the implementation of the newly expanded GGR, several donor governments provided stopgap funding to non-signing organizations,[3] which may have mitigated potential negative impacts of the policy during its first year. It is important to note that this stopgap funding is not a solution to the GGR-related loss of U.S. funds; non-U.S. donor governments have not completely filled the gap, and there is no guarantee that the current level of stopgap funding is sustainable.

Change in service delivery is the first step in our hypothetical causal pathway that could ultimately lead to negative SRH outcomes among women (Fig 1). Results from the study evaluating the Bush administration’s GGR in Ghana support this hypothesis, finding decreases in contraceptive provision, reduced contraceptive use, and increased rates of unintended pregnancy.[5] Due to the narrow window of time that the Trump administration’s policy has been in effect, this study only investigated impacts at the service delivery level. In order to detect changes in SRH outcomes among women, sufficient time is needed between the implementation of the policy and measurement of the outcomes. To this point, the Jones et al. analysis used the 2008 Ghana DHS, which was fielded seven years after the Bush administration’s GGR was implemented.[5] Future research in Uganda is needed to examine changes in modern contraceptive use, unintended pregnancy, and induced abortion rates after more time has passed since the expanded GGR’s reinstatement.

### Limitations

This study has several limitations. A more robust measure of exposure would have included changes in U.S. government FP funding flows across geographic regions in Uganda, for which detailed data were not available at the time of this study. Our exposure variable measures proxies for this change (i.e., reduced mobile outreach, facility closures, and program discontinuation among non-signing NGOs). However, GGR-related funding changes may have impacted service delivery in ways not apparent in meetings with stakeholders. In addition, NGO service provision may be concentrated in areas that are more under-resourced, resulting in selection bias in the exposure variable, complicating our quasi-experimental difference-in-difference analysis. While our exposure groups were relatively balanced at baseline, and we attempted to correct for this by controlling for district-level mCPR and IUD provision, there may still be important unmeasured differences between our “more” and “less” exposed communities that biased the results of this analysis.

The GGR may be affecting aspects of SRH service delivery at the country level, regardless of district-level fluctuations. In this case, this study may have either underestimated the true impact of the GGR or been unable to detect country-level homogenous effects. In an attempt to further investigate this limitation, we re-ran the models without the exposure variable and estimated the simple pre-post period differences in our study outcomes (see S2 Table). Across the entire sample, we observed a statistically significant decrease over time in both the proportion of facilities that received any mobile outreach visits as well as the total number of visits reported by each facility. This descriptive result, coupled with the fact that funding for a mobile outreach program was partially cut as a result of the GGR, suggests that the organizational responses described above did not sufficiently mitigate the impact of the policy on mobile outreach visits overall. However, without an appropriate comparison group, we are unable to attribute this change directly to the GGR. Changes in other outcomes from pre- to post-GGR were small in magnitude and not statistically significant.

In addition, the PMA2020 SDP sample primarily comprises public facilities. The GGR has a greater potential to impact services in private facilities, as GGR restrictions do not apply to U.S. funds directly given to foreign governments.[44] Public facilities are still impacted by the loss of U.S. funds to foreign NGOs, as these monies are used for programs that provide technical assistance, mobile outreach, and other services to public facilities. However, the lack of statistically significant impacts may be due to the relatively small proportion of private facilities in the sample.

Further, this analysis was limited to outcomes that have continually been included in the PMA2020 SDP survey, limiting our ability to capture the full scope of the policy’s impact. Potentially informative outcomes include more detailed measures regarding mobile outreach, misoprostol provision, receipt of NGO support, and caseload data. For example, while the proportion of facilities offering IUDs did not change as a result of the policy, program reductions and staffing changes may have caused the total number of IUDs provided to have decreased in the more exposed districts. Future analyses of caseload data or women’s actual use of IUDs and other family planning methods are needed to fully understand the impact of the policy on contraceptive use.

Finally, PMA2020 refreshed EAs after the 2016 survey round, so we were only able to include one year of baseline data. Having multiple years of baseline and/or follow-up data would have allowed for a greater ability to predict the outcome trajectories for the treatment and control groups, which would have led to a more robust estimation of the impact of the GGR. Without these additional years of data, this study must assume that there is no dynamic structure to the process that generates outcome trajectories and that the assignment of the exposure is not dependent on pre-2017 outcome values.[45]

## Conclusions

This study provides valuable evidence of the early impact of the newly expanded GGR in Uganda. The only statistically significant impact observed was on engagement with CHWs, which could result in negative SRH outcomes among women. The lack of significant impacts for other SRH service delivery outcomes may reflect the GGR’s limited impact, the provision of stopgap funding by other donor governments, or the resiliency of organizations to respond to changes in major funding sources. However, one must consider the timeline of this study’s implementation in interpreting these results. The true impact of this policy may not be felt for years, as stopgap funding potentially ebbs and SDPs continue to adapt to the new funding environment. Future research is needed to further assess the impact among SDPs in Uganda and among women themselves.

## Supporting information

S1 Table Difference-in-differences estimates for GGR impact one year after baseline assessment, 2018. (DOCX)

S2 Table Trends in key outcomes over time (pre to post period), controlling for study covariates. (DOCX)
