# Supplementary figures and images for: Investigating the early impact of the Trump Administration’s Global Gag Rule on sexual and reproductive health service delivery in Uganda
Source: PLoS One. 2020 Apr 28;15(4):e0231960. doi: 10.1371/journal.pone.0231960 (PMC7188216; doi:10.1371/journal.pone.0231960)

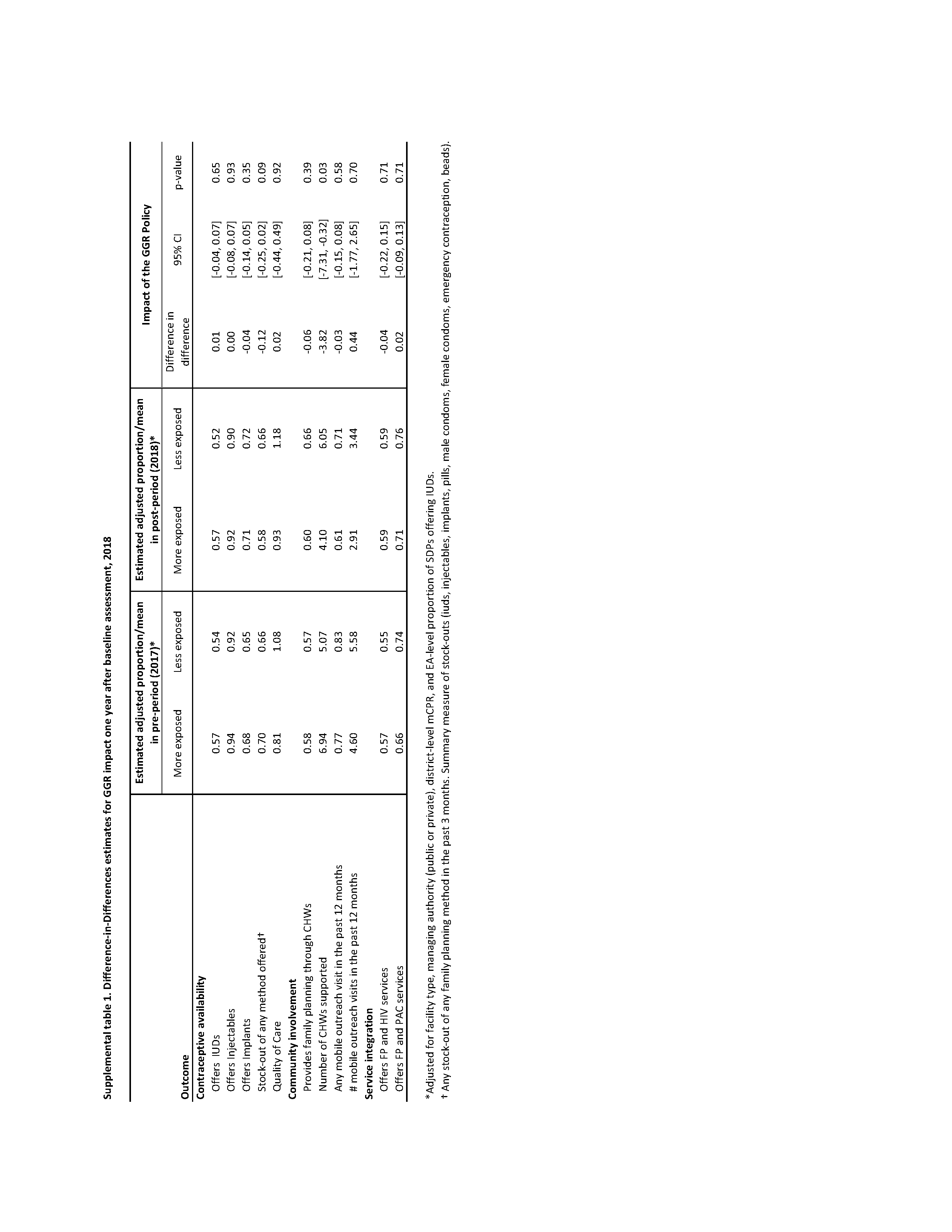

Supplement: S1 Table — (DOCX) [file pone.0231960.s001.docx]
