# Supplementary material for: Investigating the early impact of the Trump Administration’s Global Gag Rule on sexual and reproductive health service delivery in Uganda
Source: PLoS One. 2020 Apr 28;15(4):e0231960. doi: 10.1371/journal.pone.0231960 (PMC7188216; doi:10.1371/journal.pone.0231960)
Supplement: Supplementary file 2 [file pone.0231960.s002.docx]

Supplmental Table 2. Trends in key outcomes over time (pre to post period), controlling for study covariates

Outcome	Estimated adjusted proportion/mean in pre-period (2017)*	Estimated adjusted proportion/mean in post-period (2018)*	Difference (pre to post)	95% CI	p-value
**Contraceptive availability**					
Offers IUDs	0.55	0.54	-0.01	[-0.04, 0.02]	0.604
Offers Injectables	0.93	0.91	-0.02	[-0.06, 0.02]	0.289
Offers Implants	0.66	0.71	0.05	[-0.01, 0.10]	0.135
Stock-out of any method offered†	0.68	0.62	-0.06	[-0.13, 0.01]	0.089
Quality of Care	0.95	1.05	0.11	[-0.13, 0.34]	0.363
**Community involvement**					
Provides family planning through CHVs	0.57	0.63	0.05	[-0.02, 0.12]	0.148
Number of CHVs supported	6.03	5.11	-0.92	[-2.74, 0.90]	0.324
Any mobile outreach visit in the past 12 months	0.80	0.66	-0.14	[-0.19, -0.08]	<0.001
Number of mobile outreach visits in the past 12 months	5.07	3.17	-1.90	[-2.98, -0.82]	0.001
**Service integration**					
Offers FP and HIV services	0.56	0.59	0.03	[-0.06, 0.12]	0.537
Offers FP and PAC services	0.69	0.73	0.04	[-0.02, 0.09]	0.158

*Adjusted for facility type, managing authority (public or private), district-level mCPR, and EA-level proportion of SDPs offering IUDs

† Any stock-out of any family planning method in the past 3 months. Summary measure of stock-outs (iuds, injectables, implants, pills, male condoms, female condoms, emergency contraception, beads).
